# Research Progress on Benzimidazole Fungicides: A Review

**DOI:** 10.3390/molecules29061218

**Published:** 2024-03-08

**Authors:** Song Bai, Miaohe Zhang, Shouying Tang, Miao Li, Rong Wu, Suran Wan, Lijun Chen, Xian Wei, Feifei Li

**Affiliations:** 1 Guizhou Industry Polytechnic College, Guiyang 550008, China; lmiaoooo@163.com (M.L.); wurong199706@163.com (R.W.); wansuran1213@163.com (S.W.); 2 School of Chemical Engineering, Guizhou Institute of Technology, Guiyang 550003, China; zmh_870927@163.com (M.Z.); clj102128@163.com (L.C.); weixian424@sohu.com (X.W.); flylee5230@163.com (F.L.); 3 National Key Laboratory of Green Pesticide, Key Laboratory of Green Pesticide and Agricultural Bioengineering, Ministry of Education, Guizhou University, Guiyang 550025, China

**Keywords:** benzimidazole fungicides, carbendazim, toxicity, disease prevention and control, determination methods

## Abstract

Benzimidazole fungicides are a class of highly effective, low-toxicity, systemic broad-spectrum fungicides developed in the 1960s and 1970s, based on the fungicidal activity of the benzimidazole ring structure. They exhibit biological activities including anticancer, antibacterial, and antiparasitic effects. Due to their particularly outstanding antibacterial properties, they are widely used in agriculture to prevent and control various plant diseases caused by fungi. The main products of benzimidazole fungicides include benomyl, carbendazim, thiabendazole, albendazole, thiophanate, thiophanate-methyl, fuberidazole, methyl (1-{[(5-cyanopentyl)amino]carbonyl}-1*H*-benzimidazol-2-yl) carbamate, and carbendazim salicylate. This article mainly reviews the physicochemical properties, toxicological properties, disease control efficacy, and pesticide residue and detection technologies of the aforementioned nine benzimidazole fungicides and their main metabolite (2-aminobenzimidazole). On this basis, a brief outlook on the future research directions of benzimidazole fungicides is presented.

## 1. Introduction

**Figure 1:**
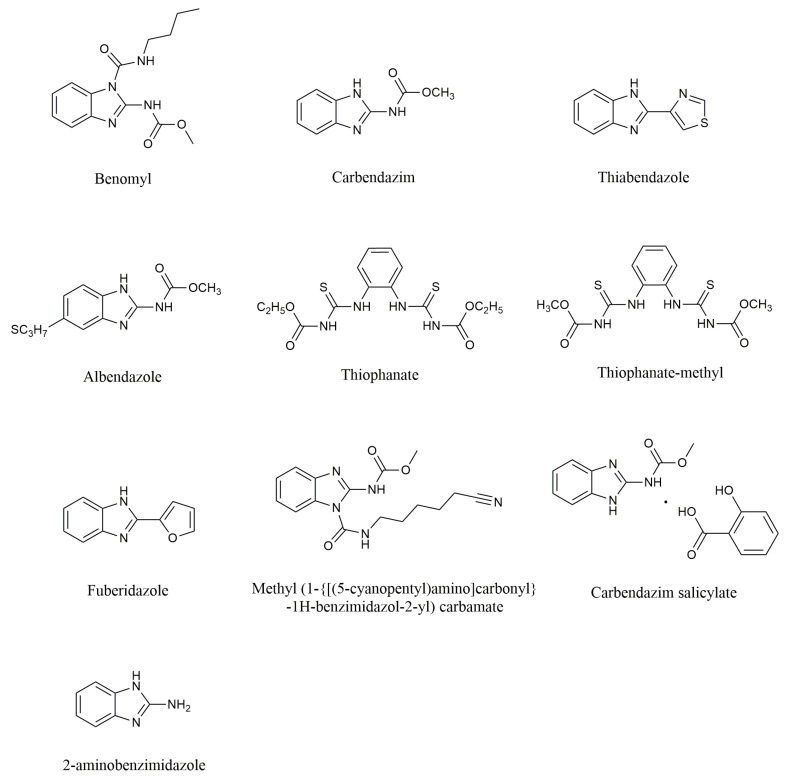
The chemical structures of benzimidazole fungicides and their main metabolite.

Benzimidazole fungicides are a class of highly effective, low-toxicity, systemic broad-spectrum fungicides based on the benzimidazole ring, which possesses fungicidal activity. The main products include benomyl, carbendazim, thiabendazole, albendazole, thiophanate, thiophanate-methyl, fuberidazole, methyl (1-{[(5-cyanopentyl)amino]carbonyl}-1*H*-benzimidazol-2-yl) carbamate, and carbendazim salicylate. Although thiophanate-methyl and thiophanate are not based on the benzimidazole ring, they are considered benzimidazole fungicides because they metabolize in plants or on their surfaces into compounds similar to carbendazim or other benzimidazole derivatives, thus exhibiting fungicidal activity [1,2,3,4]. The chemical structures of benzimidazole fungicides (benomyl, carbendazim, thiabendazole, albendazole, thiophanate, thiophanate-methyl, fuberidazole, methyl (1-{[(5-cyanopentyl)amino]carbonyl}-1*H*-benzimidazol-2-yl) carbamate, carbendazim salicylate) and their main metabolite (2-aminobenzimidazole) are depicted in Figure 1.

Benzimidazole fungicides are a class of fungicides developed during the 1960s and 1970s [5]. Benomyl was one of the earlier benzimidazole fungicides, developed by the American company DuPont in 1967. Carbendazim is a metabolite of benomyl in plants. Thiabendazole, originally an intermediate of benomyl, was also developed by DuPont in the same year. Subsequently, the American company Merck successfully developed thiabendazole, which was one of the earliest benzimidazole pesticides to be commercialized and has been in use for several decades [6]. Thiophanate-methyl was originally developed by the Japanese company Sumitomo Chemical Co., Ltd., Tokyo, Japan. Its primary mode of action is to interfere with the formation of fungal hyphae [7]. In plants, thiophanate-methyl is first converted to carbendazim, which affects the division of fungal cells, causing the germ tubes that sprout from spores to become deformed and thereby killing the fungi [3,4,8,9]. Thiophanate is quickly converted into ethyl carbendazim when sprayed on plants, which causes abnormal distortion of the germinating bud tube of pathogenic bacteria spores and distortion of the cell wall, affecting the formation of attachment cells. Thiophanate is characterized by high efficiency and low toxicity and a long residual period; it also promotes plant growth. Despite being used for several decades, benzimidazole fungicides continue to be widely used today due to their broad-spectrum and highly effective fungicidal activity.

Benzimidazole fungicides possess potent biological activity and are capable of killing a wide range of pathogenic microorganisms. They are used for anticancer, antibacterial, and antiparasitic purposes [10,11]. Among their applications, due to their particularly prominent antibacterial activity, they are extensively utilized in the agricultural sectors of grains, fruits, and vegetables to protect crops from pathogenic microorganisms, thereby enhancing crop yield and quality. At present, there is no accurate conclusion at home and abroad about the bactericidal mechanism of benzimidazole fungicides, and further research is needed. However, there are three claims. First, some researchers believe that benzimidazole fungicides are similar to the chemical structure of nucleic acid bases in the body of fungi, which can replace the bases of nucleotides and prevent the polymerization of nucleotides into nucleic acids, thus affecting the synthesis of nucleotides in fungi [12]. Secondly, benzimidazole fungicides interfere with the formation of the spindle in the mitotic process of fungi, affecting cell division, thus inhibiting the reproduction of pathogens [13]. Third, benzimidazole fungicides inhibit the segregation and elongation of gray mold mycelium, thereby preventing microtubule proteins from forming microtubules and other reticular structures, which in turn affects cell division [14]. In addition, there are researchers who believe that the bactericidal mechanism of benzimidazole fungicides is mainly through the combination of microtubule proteins with spindle fibers, which inhibits the bud cell division of the fungus, impedes the formation of spindle fibers in the process of cell mitosis, and effectively inhibits the reproduction and growth of the pathogenic bacteria to achieve antimicrobial effects [15,16,17]. These pesticides are convenient to use, exhibit no significant toxicity or side effects on crops, and have a minimal environmental impact, making them favorable agricultural fungicides with promising application prospects. However, benzimidazole fungicides face the issue of strong resistance due to their single target site of action, posing a serious threat to the control of crop diseases in the field, with carbendazim resistance being particularly notable [18]. To delay or manage pathogen resistance to benzimidazole fungicides, studies suggest mixing or rotating these agents with other fungicides with different mechanisms of action [19]. The current research on benzimidazole fungicides is primarily focused on carbendazim, thiabendazole, benomyl, and thiophanate-methyl. Research on other benzimidazole fungicides is relatively scarce and not sufficiently in-depth, indicating that these fungicides warrant further development and use. This article primarily provides a comprehensive review of the physicochemical properties, disease control efficacy, toxicological characteristics, and pesticide residue and detection techniques for benzimidazole fungicides (such as benomyl, carbendazim, thiabendazole, albendazole, thiophanate, thiophanate-methyl, fuberidazole, methyl (1-{[(5-cyanopentyl)amino]carbonyl}-1*H*-benzimidazol-2-yl) carbamate, carbendazim salicylate) and their main metabolite (2-aminobenzimidazole). Based on this information, this paper offers a prospective outlook on the future research directions for benzimidazole fungicides, which may facilitate the further development and rational utilization of these compounds.

## 2. Physical and Chemical Properties

Benzimidazole fungicides are a class of fungicides based on the benzimidazole structure, typically appearing as white or yellow crystalline powders with melting points ranging from 200 to 300 °C. They are prone to hydrolysis during dissolution. Benzimidazole fungicides are usually basic or neutral, insoluble in most organic solvents and pure water substances, but most of them can dissolve in strong polar solvents such as dimethyl sulfoxide and *N*,*N*-dimethylformamide and in strong acids. The imidazole ring often found in the molecules of benzimidazole fungicides is a highly reactive structure. Various fungicides with different effects and properties can be synthesized by substituting at different positions on the imidazole ring. Benzimidazole fungicides (such as benomyl, carbendazim, thiabendazole, albendazole, thiophanate, thiophanate-methyl, fuberidazole, methyl (1-{[(5-cyanopentyl)amino]carbonyl}-1*H*-benzimidazol-2-yl) carbamate, and carbendazim salicylate) and their main metabolite (2-aminobenzimidazole) have physicochemical properties as shown in Table 1.

Carbendazim has stable chemical properties, and the technical material can be stored for 2 to 3 years in a cool and dry place. The pure product is a light-gray or beige powder with a melting point of 302–307 °C. At 24 °C, its solubility in water is 0.008 g/L, in dimethylformamide is 5 g/L, in acetone is 0.3 g/L, in ethanol is 0.3 g/L, in chloroform is 0.1 g/L, and in ethyl acetate is 0.135 g/L. It hydrolyzes slowly in an alkaline medium and is stable in an acidic medium, capable of forming salts. Thiabendazole pure product is a white crystalline powder, odorless and tasteless, stable in water, acid, and alkaline solutions. The melting point is 298–301 °C. At 20 °C, its solubility in water is 0.03 g/L, in acetone is 2.43 g/L, in methanol is 8.28 g/L, in xylene is 0.13 g/L, in ethyl acetate is 1.49 g/L, and in octanol is 3.91 g/L. The pure product of benomyl is a white crystalline powder with a melting point of 140 °C (decomposition). It is insoluble in water and oil but soluble in chloroform, acetone, and dimethylformamide. The pure product of albendazole is a white powder with a melting point of 206–212 °C. It is odorless and tasteless, insoluble in water, slightly soluble in ethanol, chloroform, hot dilute hydrochloric acid, and dilute sulfuric acid, and soluble in glacial acetic acid. Thiophanate is a colorless flaky crystal with a melting point of 195 °C (decomposition). It is insoluble in water but soluble in organic solvents such as dimethylformamide, acetonitrile, and cyclohexane. It can recrystallize in solvents like ethanol and acetone, and it has stable chemical properties. The pure product of thiophanate-methyl is a colorless crystalline solid, while the technical powder (93%) appears as yellow crystals. It has a melting point of 172 °C (decomposition). Its solubility in water and other solvents is very low, but it dissolves easily in dimethylformamide, dioxane, and chloroform and is also soluble in solvents such as acetone, methanol, ethanol, and ethyl acetate. It is stable in the presence of acids and bases. The pure form of fuberidazole is a colorless crystalline solid with a melting point of 286 °C. At room temperature, it is insoluble in water but readily soluble in organic solvents such as methanol, ethanol, and acetone. Its solubility in water is 0.0078 g/L; in dichloromethane, it is 1 g/L; and in isopropanol, it is 5 g/L. Additionally, it is also soluble in methanol, ethanol, and acetone. It is unstable when exposed to light. The pure form of methyl (1-{[(5-cyanopentyl)amino]carbonyl}-1*H*-benzimidazol-2-yl) carbamate is a colorless crystal that is insoluble in water but soluble in organic solvents such as toluene, dichloromethane, dimethylformamide, and cyclohexane. The wettable powder for carbendazim salicylate is a grayish-white powder, and the soluble agent is a dark-brown liquid, both of which are soluble in water. The main metabolite of benzimidazole fungicides, 2-aminobenzimidazole, is a pure substance that appears as a white or light-yellow crystalline solid. It has a melting point of 226–230 °C and a boiling point of 235.67 °C. It is soluble in water, ethanol, and acetone but insoluble in diethyl ether and benzene.

## 3. Disease Prevention and Control

In contemporary agricultural production, the effective prevention and control of crop diseases is a key factor in ensuring food security and increasing yield. As a broad-spectrum and highly efficient fungicide, benzimidazole fungicides have significant effects in controlling a variety of crop diseases. For example, benomyl has a good inhibitory effect on diseases caused by fungi from the subphyla Ascomycotina, Deuteromycotina, and certain Basidiomycotina [20], but it is ineffective against rusts, flagella, and zygomycetes. Benomyl is widely used in agriculture to control a variety of crop diseases, including powdery mildew and scab in apples and pears, wheat scab, rice blast, cucurbit scab and anthracnose, eggplant gray mold, tomato leaf mold, scab of cucurbits, allium gray mold rot, celery gray-spot disease, asparagus stem blight, citrus scab and gray mold, soybean sclerotinia, peanut brown-spot disease, as well as sweet potato black-spot disease and dry rot [21]. The control of the disease by benzimidazole fungicides is shown in Table 2. Carbendazim is effective against diseases caused by fungi such as *Botrytis cinerea*, *Fusarium* spp., *Alternaria* spp., *Penicillium* spp., *Cladosporium* spp., *Sclerotinia* spp., *Venturia* spp., *Plasmopara* spp., *Rhizoctonia* spp., and others. These include wheat diseases such as wheat head blight, loose smut, and glume blotch; oat loose smut; cereal stem rot; powdery mildew in wheat, apples, pears, grapes, and peaches; apple scab; pear black spot; pear ring rot; grape gray mold and white rot; cotton seedling blight and boll rot; peanut leaf spot and root rot; tobacco anthracnose; tomato early blight and gray mold; pineapple disease in sugarcane; beet leaf spot; rice blast, sheath blight, and sesame leaf spot, among others. However, despite its effectiveness against certain pathogens of the Ascomycota subphylum and most pathogens of the Deuteromycota subphylum, it is ineffective against oomycetes and bacteria [21]. Thiabendazole exhibits good antimicrobial effects against the main pathogens in ascomycetes, basidiomycetes, and deuteromycetes. However, it is ineffective against pathogens belonging to genera such as Mucor, Peronospora, Phytophthora, Rhizopus, and Pythium, and it does not show activity against oomycetes and zygomycetes causing smut diseases [21]. Thiabendazole is primarily used for the prevention and treatment of various plant diseases, including citrus green mold, blue mold, and stem-end rot, and is also suitable for post-harvest preservation [21]. Albendazole is effective against diseases caused by various basidiomycetes and ascomycetes, such as rice blast and tobacco anthracnose [21]. Thiophanate can effectively prevent and treat a variety of plant diseases, including Fusarium head blight (scab), powdery mildew, and smut in cereal crops; rice blast, sheath blight, and kernel smut in rice; as well as various fungal diseases in vegetables, fruits, and other crops [21,22]. Thiophanate-methyl can control and prevent diseases such as rice blast and sheath blight in rice, rust and powdery mildew in wheat, Fusarium head blight (scab) and smut in cereal crops, sclerotinia in rapeseed, downy mildew in tomatoes, anthracnose and leaf spot in various vegetables, scab in peanuts, as well as powdery mildew and anthracnose in fruit trees [21,22]. Fuberidazole exhibits effective antimicrobial properties against major pathogens from Ascomycetes, Basidiomycetes, and Deuteromycetes, and is suitable for the prevention and control of wheat diseases such as black head mold and snow mold [21,22]. Methyl (1-{[(5-cyanopentyl)amino]carbonyl}-1*H*-benzimidazol-2-yl) carbamate can prevent and treat diseases such as rice bakanae disease, and powdery mildew in apples and pears [21], whereas carbendazim salicylate can effectively control a variety of fungal diseases such as wheat scab and cotton wilt [21]. These agents provide a diversified selection to meet the disease management needs of different crops.

## 4. Toxicological Properties

According to the Chinese pesticide toxicity classification standards (Table 3) [23,24,25,26], benzimidazole fungicides such as benomyl, carbendazim, thiabendazole, and carbendazim salicylate are considered low-toxicity or slightly toxic pesticides. Pesticides like thiophanate-methyl, thiophanate, fuberidazole, and methyl (1-{[(5-cyanopentyl)amino]carbonyl}-1*H*-benzimidazol-2-yl) carbamate are classified as moderately low-toxicity or low-toxicity pesticides. The main metabolite of benzimidazole fungicides, 2-aminobenzimidazole, is of low toxicity. Benzimidazole fungicides possess systemic properties, allowing them to be absorbed into the flesh of agricultural products while killing harmful organisms. The potential dosage present in the produce can cause chronic or acute toxicity to humans, posing a risk to human health [27]. Benzimidazole fungicides have teratogenic and embryotoxic effects on a variety of animals [28,29,30,31]. The toxicity of benzimidazole fungicides is shown in Table 4. Studies have shown that carbendazim has certain toxicity to humans and animals and can cause symptoms of poisoning such as excitement, convulsions, mental confusion, nausea and vomiting, dizziness and headache, chest tightness, and upper abdominal tenderness [32,33]. It can also cause liver diseases, as well as chromosomal abnormalities, endocrine system disorders, and teratogenic effects [34,35]. Thiabendazole has a certain level of toxicity to humans, primarily affecting the liver, nervous system, and bone marrow [36,37]. The main metabolite of benzimidazole fungicides, 2-aminobenzimidazole, is toxic to human skin and eyes [38]. Based on these findings, countries around the world have imposed strict controls on the use of benzimidazole fungicides, and there are stringent requirements for their residue levels. The standards for pesticide residue limits in China [39], South Korea [40], and the European Union [41], as well as the acceptable daily intake (ADI) standards in China and South Korea, are shown in Table 5.

## 5. Pesticide Residue and Detection Technology

Benzimidazole fungicides are extensively employed during the growth and storage phases of a variety of crops, including vegetables and fruits, for the control and prevention of fungal diseases. Improper use can result in residues remaining in fruits and vegetables. With the continuous advancement of science and technology, methods for detecting pesticide residues have also been evolving. Detection techniques have progressed from the earliest paper chromatography, thin-layer chromatography, and gel permeation chromatography to current methods such as spectroscopic analysis [42], immunoassay, gas chromatography (GC) [43], gas chromatography–mass spectrometry (GC-MS) [44], high-performance liquid chromatography (HPLC) [45], and liquid chromatography–mass spectrometry (LC-MS) [46]. Currently, in the residual analysis of benzimidazole fungicides reported both domestically and internationally, the focus is primarily on the study of carbendazim and thiabendazole. The detection methods commonly employed are HPLC and liquid chromatography–tandem mass spectrometry (LC-MS/MS) [47,48,49]. Sample pretreatment techniques include liquid–liquid extraction (LLE), solid-phase extraction (SPE), supercritical fluid extraction (SFE), and microwave-assisted extraction (MAE). With the widespread adoption of the QuEChERS method, which is rapid, simple, inexpensive, effective, reliable, and safe, it is now commonly used for high-efficiency and rapid pretreatment of samples [50,51,52].

### 5.1. Spectroscopic Analysis Method

Spectroscopic analysis encompasses methods such as ultraviolet spectrophotometry, fluorescence analysis, phosphorescence, cryoluminescence, and other luminescence analytical techniques. Among these, fluorescence analysis is the most commonly employed, including both fluorescence excitation and emission spectroscopy. Compared to ultraviolet absorption spectrophotometry, fluorescence analytical techniques are extensively applied in the residual analysis of benzimidazole fungicides in environmental and food samples due to their higher sensitivity and the advantage of being rapid and straightforward. Zhong et al. [38] employed three-dimensional fluorescence spectroscopy in conjunction with chemometric second-order calibration for the quantitative analysis of thiram and carbendazim in red wine. By utilizing direct quantitative analysis, the method is characterized by short analysis time and high efficiency in extraction and separation. Subhani et al. [53] employed an ion chromatography system coupled with a post-column photochemical derivatization fluorescence detector for the detection of carbendazim. Under optimized conditions, the method exhibited a strong linear response with correlation coefficients greater than or equal to 0.9966, demonstrated good reproducibility, and achieved high recovery rates.

### 5.2. Gas Chromatography Technology

In the 1970s, gas chromatography made significant contributions to the enhancement of pesticide residue detection techniques due to its high separation efficiency and sensitivity. Gas chromatography employs an inert gas as the mobile phase to separate and identify substances and is suitable for the analysis of volatile and chemically stable compounds. However, it is not appropriate for pesticides with high boiling points and thermal instability. For example, carbendazim, a benzimidazole fungicide, is a thermally unstable compound and is not suitable for direct analysis by gas chromatography. However, Xu et al. [43] established a derivatization gas chromatography–nitrogen–phosphorus detection method for the determination of carbendazim residues in environmental water samples, which has the characteristics of high accuracy, good reproducibility, and low detection limit. Mass spectrometry is widely used as a detector because of its high resolution and accuracy. Gas chromatography–mass spectrometry (GC-MS) combines the high-efficiency separation capability of chromatography with the accurate identification ability of mass spectrometry. This not only enhances the separation efficiency but also improves the accuracy of identification, achieving the goals of both qualitative and quantitative analysis. Zhang et al. [54] detected the residue of thiabendazole in longan samples by gas chromatography, which is a simple, sensitive, and accurate method, and it is a more ideal method for the detection of thiabendazole residue in longan.

### 5.3. High-Performance Liquid Chromatography Technology

High-performance liquid chromatography (HPLC) is an analytical detection technique developed in the 1970s and is one of the most commonly used analytical methods. Currently, 80% of compounds require separation and detection by high-performance liquid chromatography. In national standards, the detection and analysis of carbendazim, thiabendazole, thiram, and benomyl are carried out using reverse-phase high-performance liquid chromatography. For example, studies have reported the detection of carbendazim residues in edible mushrooms, soybeans, wine, rapeseed, asparagus, American ginseng herbs, dendrobium, longan, and fruit juices by HPLC [55,56,57]. Caprioli et al. [58] employed HPLC with a diode-array detector (DAD) to detect the residues of thiabendazole and propiconazole in bovine liver. Al-Ebaisat [59] utilized a combination of high-performance liquid chromatography and solid-phase extraction to measure the levels of carbendazim and benomyl in tomato paste. The recovery rate was 90.0–95.5%, with a detection limit of 5 μg/kg. Liang et al. [60] utilized matrix solid-phase dispersion (MSPD) combined with dispersive liquid–liquid microextraction (DLLME), which was optimized by chemometric methods. They applied this optimized method (MSPD-DLLME-HPLC-UV) for the detection of carbendazim, thiabendazole, and thiophanate in vegetable samples and achieved effective extraction, purification, and quantification of these substances. The method showed good linearity in the concentration range of 0.25–5 μg/g, and the recoveries were in the range of 65.4–124.0%. In addition, with the widespread use of mass spectrometry detectors, liquid chromatography–mass spectrometry (LC-MS) has also been widely used for the determination of benzimidazole fungicides. The principle of LC-MS involves initially ionizing the sample to be analyzed and then separating it according to the mass-to-charge ratio to obtain a mass spectrum. By using the mass spectral information of the sample and its fragments, qualitative and quantitative analysis of the test sample can be conducted. It is an important method for determining organic compounds. Wu et al. [61] used LC-MS to detect the residues of albendazole in watermelon, achieving an average recovery rate of 82.3–93.6%. Similarly, employing LC-MS technology, Kim et al. [62] detected residues of carbendazim and thiabendazole in bean sprouts, with recovery rates ranging from 89.5% to 103.2% when the added concentration was 20–40 mg/kg. Blasco et al. [63] applied LC-MS techniques to determine the content of carbendazim in peaches and nectarines. Bean et al. [64] used a G-protein immunoadsorbent column coupled with reverse-phase LC-MS to detect carbendazim in lake water and soil samples. The advantage of this method is that it utilizes immunoadsorbent extraction technology, which can reduce matrix interference and increase the accuracy of sample detection. Wang [65] determined carbendazim in orange juice using QuEChERS and liquid chromatography–mass spectrometry/mass spectrometry (LC-MS/MS). Furthermore, since benomyl is very unstable and easily decomposes into carbendazim when exposed to organic solvents or water, Dong et al. [66] employed the QuEChERS method for extraction and HPLC-MS/MS for the determination of thiabendazole and carbendazim in watercress, with an average recovery rate of 96.2–102.9%. The limit of quantification was 0.01 mg/kg.

### 5.4. Enzyme-Linked Immunosorbent Assay

The enzyme-linked immunosorbent assay (ELISA) is a technique in which an antigen or antibody is linked to an enzyme to form an enzyme-labeled antigen or antibody complex. Upon the addition of a substrate, the enzyme catalyzes a reaction that produces a colored substance. The intensity of the color is directly proportional to the amount of the product, allowing for qualitative and quantitative analysis based on the shade of the color. This technique offers advantages such as speed, sensitivity, and ease of standardization [67]. For example, the researchers designed and synthesized the hapten of carbendazim based on its molecular structure and specific functional groups, prepared artificial antigens and antibodies, and established and optimized a method for the indirect competitive ELISA for the quantitative detection of carbendazim [68]. This provides a foundation for the development of test kits suitable for the rapid, on-site detection of pesticide residues in fruits and vegetables. Šmídová et al. [69] proposed an immunoassay for the detection of thiabendazole residues in food, with a detection limit of 0.005 mg/kg in apple juice and a detection limit of 0.05 mg/kg in pears and oranges. Blažková et al. [70] established a rapid detection method for thiabendazole residues in fruit juices based on a strip-based immunoassay. Using carbon nanoparticles as markers, the non-direct competitive immunoassay for thiabendazole can be completed in 10 min under optimal conditions. The method is precise, stable, and specific, with a detection limit of 0.25 ppb and a recovery rate of 89.9 to 123.6%. Estevez et al. [71] developed a sensitive and specific competitive immunoassay method based on surface plasmon resonance (SPR) for the detection of thiabendazole in oranges. They used an indirect assay format by immobilizing the thiabendazole–protein conjugate on a gold film surface. The analyte from the oranges was extracted with methanol, achieving satisfactory recovery rates. The sensitivity and reliability tests on orange samples confirmed the effectiveness and dependability of the SPR sensor, making it suitable for determining thiabendazole in food products.

## 6. Conclusions

Benzimidazole fungicides occupy an important position in the field of agricultural disease prevention and control due to their broad-spectrum antifungal activity. The current research on benzimidazole fungicides is mainly focused on carbendazim, thiabendazole, benomyl, and thiophanate-methyl, and the antifungal spectrum of these compounds is broader and more effective. Pesticide residue detection often uses liquid chromatography and liquid chromatography–tandem mass spectrometry. With the changes in crop diseases and considerations for environmental safety and food safety, the development of benzimidazole fungicides is showing new trends. Future research will focus more on the development of high-efficiency, low-toxicity, low-residue, environmentally friendly green pesticides. At the same time, due to the serious resistance issues of benzimidazole fungicides, subsequent research will further address the resistance problems of these fungicides. Finally, benzimidazole fungicides can be used in combination with intelligent pesticide application technologies, which will make the use of benzimidazole fungicides more precise, thereby enhancing their application value and environmental safety in agricultural production.

## Figures and Tables

**Table 1:** Physicochemical properties of benzimidazole fungicides and their main metabolite.

Compounds	Chemical Formula	Molecular Mass	Melting Points	Solubility	Properties	CAS Number
Benomyl	C_14_H_18_N_4_O_3_	290.318	140 °C	Insoluble in water and oil; soluble in chloroform, acetone, dimethylformamide	White crystalline powder	17804-35-2
Carbendazim	C_9_H_9_N_3_O_2_	191.187	302–307 °C	At 24 °C, slightly soluble in water; soluble in dimethylformamide, acetone, ethanol, chloroform, ethyl acetate	Light-gray or beige powder	10605-21-7
Thiabendazole	C_10_H_7_N_3_S	201.25	298–301 °C	At 20 °C, slightly soluble in water; soluble in acetone, methanol, xylene, ethyl acetate, octanol	White crystalline powder	148-79-8
Albendazole	C_12_H_15_N_3_O_2_S	265.33	206–212 °C	Insoluble in water; slightly soluble in ethanol, chloroform, hot dilute hydrochloric acid, dilute sulfuric acid; soluble in glacial acetic acid	White powder	54965-21-8
Thiophanate	C_14_H_18_N_4_O_4_S_2_	370.447	195 °C	Insoluble in water; soluble in dimethylformamide, acetonitrile, cyclohexane, ethanol, acetone, and other organic solvents	Colorless flaky crystals	23564-06-9
Thiophanate-methyl	C_12_H_14_N_4_O_4_S_2_	342.39	172 °C	Slightly soluble in water; soluble in dimethylformamide, dioxane, chloroform, acetone, methanol, ethanol, ethyl acetate, and so on	Colorless crystalline solid	23564-05-8
Fuberidazole	C_11_H_8_N_2_O	184.19	286 °C	At room temperature, insoluble in water; soluble in methanol, ethanol, acetone, dichloromethane, isopropanol, and other organic solvents	Colorless crystalline solid	3878-19-1
Methyl (1-{[(5-cyanopentyl)amino]carbonyl}-1*H*-benzimidazol-2-yl) carbamate	C_16_H_19_N_5_O_3_	329.354	/	Insoluble in water; soluble in toluene, dichloromethane, dimethylformamide, cyclohexane, and other organic solvents	Colorless crystals	28559-00-4
Carbendazim salicylate	C_16_H_15_N_3_O_5_	329.28	/	Soluble in water	Deep-brown liquid (soluble solvent), grayish-white powder (wettable powder)	/
2-Aminobenzimidazole	C_7_H_7_N_3_	133.15	226–230 °C	Soluble in water, ethanol, acetone; insoluble in ether, benzene	White or pale-yellow crystalline solid	934-32-7

‘/’ indicates there is no relevant report.

**Table 2:** Benzimidazole fungicides main disease control.

Compounds	Disease Control
Benomyl	Apples (powdery mildew, scab), pears (powdery mildew, scab), wheat (wheat scab), rice (rice blast), cucurbits (cucurbit scab, anthracnose), eggplant (eggplant gray mold), tomatoes (tomato leaf mold), allium (allium gray-mold rot), celery (celery gray-spot disease), asparagus (asparagus stem blight), citrus (scab, gray mold), soybean (soybean sclerotinia), peanut (peanut brown-spot disease), sweet potato (black-spot disease, dry rot) [21]
Carbendazim	Wheat (head blight, loose smut, glume blotch), cereal (stem rot), apples (powdery mildew, scab), pears (powdery mildew, black-spot, ring rot), grape (powdery mildew, gray mold, white rot), peach (powdery mildew), cotton (cotton seedling blight, boll rot), peanut (peanut leaf spot and root rot), tobacco (tobacco anthracnose), tomatoes (early blight, gray mold), sugarcane (pineapple disease), beet (beet leaf spot), rice (rice blast, sheath blight), sesame (sesame leaf spot) [21]
Thiabendazole	Citrus (citrus green mold, blue mold, stem-end rot) [21]
Albendazole	Rice (rice blast), tobacco (anthracnose) [21]
Thiophanate	Cereal crops (Fusarium head blight, powdery mildew, smut), rice (rice blast, sheath blight, kernel smut) [21,22]
Thiophanate-methyl	Rice (rice blast, sheath blight), wheat (rust, powdery mildew), cereal crops (Fusarium head blight, smut), rapeseed (sclerotinia), tomatoes (downy mildew), vegetables (anthracnose, leaf spot), peanut (scab), fruit trees (powdery mildew, anthracnose) [21,22]
Fuberidazole	Wheat (black head mold, snow mold) [21,22]
Methyl (1-{[(5-cyanopentyl)amino]carbonyl}-1*H*-benzimidazol-2-yl) carbamate	Rice (rice bakanae disease), apples (powdery mildew), pears (powdery mildew) [21]
Carbendazim salicylate	Wheat (wheat scab), cotton (cotton wilt) [21]

**Table 3:** Pesticide product toxicity classification standards.

Toxicity Level	Oral LD_50_ (mg/kg)	Dermal LD_50_ (mg/kg)	LD_50_ Inhalation (mg/m^3^)
Extremely Toxic	≤5	≤20	≤20
Highly Toxic	>5~50	>20~200	>20~200
Moderately Low Toxicity	>50~500	>200~2000	>200~2000
Low Toxicity	>500~5000	>2000~5000	>2000~5000
Slightly Toxic	>5000	>5000	>5000

**Table 4:** Toxicological properties of benzimidazole fungicides and their main metabolites [21].

Compounds	Toxicological Properties
Benomyl	Rat acute oral LD_50_ > 5000 mg/kg; rabbit acute dermal LD_50_ > 5000 mg/kg; temporary irritant to rabbit eyes; no abnormalities found in dogs fed with 500 mg/kg for two years; non-toxic to earthworms.
Carbendazim	Rat acute oral LD_50_ > 15,000 mg/kg; rat acute percutaneous LD_50_ > 2000 mg/kg; rabbit acute oral LD_50_ > 10,000 mg/kg; no irritation to rabbit’s eyes and skin; no abnormality was found when feeding 300 mg/kg to dogs for two years; and it is non-toxic to earthworms.
Thiabendazole	Rat acute oral LD_50_ > 3100 mg/kg; mouse acute oral LD_50_ > 3600 mg/kg; rabbit acute percutaneous LD_50_ > 2000 mg/kg; no irritation to rabbit eyes and skin; 40 mg/kg fed to a dog for two years without abnormalities; no teratogenicity, mutagenicity, carcinogenicity to animals.
Albendazole	Rat acute oral LD_50_ > 4287 mg/kg; rat acute percutaneous LD_50_ > 608 mg/kg; mouse acute oral LD_50_ > 17,531 mg/kg.
Thiophanate	Mouse acute oral LD_50_ > 15,000 mg/kg.
Thiophanate-methyl	Rat acute oral LD_50_ > 7500 mg/kg; rat acute percutaneous LD_50_ > 10,000 mg/kg; mouse acute oral LD_50_ > 17,531 mg/kg.
Fuberidazole	Rat acute oral LD_50_ > 1100 mg/kg; rat acute percutaneous LD_50_ > 1000 mg/kg.
Methyl (1-{[(5-cyanopentyl)amino]carbonyl}-1*H*-benzimidazol-2-yl) carbamate	Rat acute oral LD_50_ >2500 mg/kg; rabbit acute oral LD_50_ >1000 mg/kg; dog acute oral LD_50_ > 500 mg/kg.
Carbendazim salicylate	Rat acute oral LD_50_ > 500 mg/kg.
2-aminobenzimidazole	Rat acute oral LD_50_ > 500 mg/kg.

**Table 5:** Acceptable daily intake (ADI, mg/kg bw) and maximum residue limits (MRLs, mg/kg) for benzimidazole pesticides in foods.

Compounds	China’s ADI (mg/kg bw)	China’s MRL (mg/kg)	Korea’s ADI (mg/kg bw)	Korea’s MRL (mg/kg)	EU’s MRL (mg/kg)
Benomyl	0.1	0.5–5	0.1	0.01–20 *	0.05–2
Carbendazim	0.03	0.02–20	0.03	0.01–20 *	0.05–2
Thiabendazole	0.1	0.05–15	0.1	0.2–40	0.01–20
Albendazole	0.05	0.1–5	/	/	/
Thiophanate	/	/	/	/	/
Thiophanate-methyl	0.09	0.1–20	0.08	0.01–20 *	0.05–6
Fuberidazole	/	/	/	/	0.01–0.05
Methyl (1-{[(5-cyanopentyl)amino]carbonyl}-1*H*-benzimidazol-2-yl) carbamate	/	/	/	/	/
Carbendazim salicylate	/	/	/	/	/
2-Aminobenzimidazole	0.1	0.5–5	0.1	0.01–20	0.05–2

‘/’ indicates there is no relevant report; ‘*’ sum of benomyl, carbendazim, and thiophanate-methyl expressed as carbendazim.

## Data Availability

Not applicable.
